# Association of coronary artery calcium with heart rate variability in the Brazilian Longitudinal Study of Adult Health - ELSA-Brasil

**DOI:** 10.1590/1414-431X2023e12364

**Published:** 2023-02-27

**Authors:** R.A. Hoshi, I.S. Santos, M.S. Bittencourt, E.M. Dantas, R.V. Andreão, J.G. Mill, P.A. Lotufo, I.M. Benseñor

**Affiliations:** 1 Centro de Estudos Clínicos e Epidemiológicos do Hospital Universitário, Universidade de São Paulo, São Paulo, SP, Brasil; 2 Departamento de Ciências Biológicas, Universidade Federal do Vale do São Francisco, Petrolina, PE, Brasil; 3 Departamento de Engenharia Elétrica, Instituto Federal do Espírito Santo, Vitória, ES, Brasil; 4 Departamento de Ciências Fisiológicas, Universidade Federal do Espírito Santo, Vitória, ES, Brasil

**Keywords:** Autonomic nervous system, Atherosclerosis, Heart rate control, Vascular stiffness, Vascular calcification

## Abstract

Current data shows that the autonomic and vascular systems can influence each other. However, only a few studies have addressed this association in the general population. We aimed to investigate whether heart rate variability (HRV) was associated with coronary artery calcium (CAC) in a cross-sectional analysis of the Brazilian Longitudinal Study of Adult Health (ELSA-Brasil). We examined baseline data from 3138 participants (aged 35 to 74 years) without previous cardiovascular disease who underwent CAC score assessment and had validated HRV recordings. Prevalent CAC was defined as a CAC score>0, and HRV analyses were performed over 5-min segments. We detected CAC score>0 in 765 (24.4%) participants. Subgroup analyses in older participants (≥49 years) adjusted for sociodemographic and clinical variables revealed that CAC score>0 was associated with lower values of standard deviation of NN intervals (SDNN) (odds ratio [OR]=1.32; 95%CI: 1.05,1.65), root mean square of successive differences between adjacent NN intervals (RMSSD) (OR=1.28; 95%CI: 1.02,1.61), and low frequency (LF) (OR=1.53, 95%CI: 1.21,1.92). Interaction analysis between HRV indices and sex in age-stratified groups revealed significant effect modification: women showed increased OR for prevalent CAC in the younger group, while for men, the associations were in the older group. In conclusion, participants aged ≥49 years with low SDNN, RMSSD, and LF values were more likely to present prevalent CAC, suggesting a complex interaction between these markers in the pathogenesis of atherosclerosis. Furthermore, our results suggested that the relationship between CAC and HRV might be sex- and age-related.

## Introduction

Although coronary artery disease (CAD) is the leading cause of death throughout the world (1), a significant proportion of patients who suddenly develop clinical symptoms were not previously identified as high risk by current strategies (2). For this reason, there is a continued pursuit to identify noninvasive methods for the early detection of individuals at increased risk for atherosclerosis. Since vascular calcification is a hallmark of atherosclerosis, coronary artery calcium (CAC) scanning has emerged as the top contender among several novel screening tests to improve risk assessment of atherosclerotic cardiovascular disease (3-5).

Cardiac autonomic impairment has also been associated with an increased risk of fatal and nonfatal CAD (6,7) and can be measured by heart rate variability (HRV) analysis. The clinical significance of this measure is increasingly recognized as an important physiological marker and a diagnostic tool in the detection of autonomic impairment and prediction/prognosis of several cardiac and non-cardiac disorders (8-13). Essentially, a combination of higher values of standard HRV indices under resting conditions means an adequate coupling between the sympathetic and the parasympathetic branches of the autonomic nervous system (ANS). It is a characteristic of a more favorable cardio-autonomic profile that indirectly reflects superior control over multiple organic systems (9,12,14). In contrast, lower variability is related to cardiovascular and all-cause death (15), increased risk of adverse cardiac outcomes, and underlying risk factors such as diabetes, hypertension, and thyroid dysfunctions (10,12,13).

Although the main pathogenic factors of atherosclerotic disease come from the arterial lumen, such as inflammation (9,16,17), vascular function impairment can also originate outside the vessels. The ANS innervates vascular walls and regulates contractility and tension. Therefore, autonomic dysfunctions may exert detrimental effects on endothelial and vascular tissues, favoring atherogenesis (18). Current experimental data show that the autonomic and vascular systems can reciprocally influence each other (18,19). Therefore, investigating this interplay is valuable and would provide information that may disclose potential novel uses for these techniques in the clinical setting. The bottom line is that detecting high-risk individuals for CVD who are potential candidates for early preventive interventions is critical in health management, even in asymptomatic patients. The relationship between cardiac autonomic dysfunction and CAC represents such clinical importance that has been investigated in diabetic patients at risk for cardiovascular outcomes due to cardio-autonomic neuropathy (20,21). Nonetheless, to our knowledge, only a few studies have addressed this association in general population samples. Therefore, we hypothesized that prevalent CAC is associated with a worse cardio-autonomic profile. To test this assumption, we examined baseline HRV linear indices and CAC score from the Brazilian Longitudinal Study of Adult Health (ELSA-Brasil) cohort to explore whether there is an association between these two markers.

## Material and Methods

The ELSA-Brasil is a multi-center observational and longitudinal study that primarily aims to investigate the incidence and progression of diabetes and cardiovascular diseases and their biological, behavioral, environmental, occupational, psychological, and social factors over a long-term follow-up. Detailed design, objectives, and cohort profile have been published elsewhere (22,23). Briefly, this cohort study examined 15105 civil servants from 6 institutions in different Brazilian cities. Active or retired employees of the six institutions aged 35 to 74 years were eligible for the study. The baseline assessment included questionnaires, medical measurements, and laboratory examinations. The baseline assessment took place from August 2008 to December 2010, and the participants at the São Paulo ELSA-Brasil site were invited to perform a computed tomographic (CT) examination to quantify CAC. Approvals were granted by the Institutional Review Boards of all the centers in accordance with the Declaration of Helsinki, and all the participants signed a written informed consent form.

Besides having a CT scan for CAC evaluation (4549 participants from the São Paulo site), inclusion criteria required a validated ECG recording for HRV analysis. We excluded participants with prior CV disease (myocardial infarction, stroke, heart failure, coronary revascularization), arrhythmias (atrial fibrillation or flutter), and chronic kidney disease. We also excluded participants using beta-blockers, angiotensin-converting enzyme inhibitors, calcium channel blockers, or antiarrhythmic drugs due to the effect of these medications on heart rate control and variability.

CAC examinations were performed using a 64-detector CT scanner (Brilliance 64; Philips Healthcare, The Netherlands). An ECG-gated prospective calcium score examination with a tube potential of 120 kV and a tube current adjusted to body habitus was performed. Images were reconstructed in 2.5-mm slice thickness using standard filtered back projection. An experienced blinded cardiologist evaluated the CT images using a semiautomatic software (Calcium Scoring; Philips Workstation). CAC scores were reported in Agatston units (24). For analyses, CAC scores were dichotomized as 0 or >0 Agatston units, and we defined prevalent CAC as a CAC score >0.

Heart rate beat-to-beat interval (RRi) recordings were collected during participants' first visit to the investigation center. The protocol used to record R-R interval series and analyze HRV in the ELSA-Brasil has been published elsewhere (25). A 10-min resting-state electrocardiogram (ECG) recording was obtained in the supine position during spontaneous breathing and without task demands. The artifact detection and spectral analytic techniques were the same as those used by Dantas et al. (25), in which the R-R series were automatically preprocessed to remove ectopic beats and artifacts, and linear interpolation was employed to replace any removed beats.

HRV analyses were performed in 5-min segments from each 10-min R-R series. Time-domain analysis consisted of the standard deviation of NN interval (SDNN) and the root mean square of successive differences between adjacent NN intervals (RMSSD). Power spectral analysis was carried out by autoregressive modeling, estimated by the Yule-Walker method (26), using the Levinson-Durbin recursive algorithm to estimate directly from the data the coefficients of the AR model and the variance of the white noise. The number of coefficients (p) was chosen according to Akaike's figure of merit (26,27). Low frequency (LF, 0.04-0.15 Hz) and high frequency (HF, 0.15-0.40 Hz) power spectral bands were reported in absolute values (ms^2^) and in normalized values (n; LF/(LF+HF) and HF/(LF+HF), respectively) (28).

Computed tomography scans were scheduled on different days from the first visit to the investigation center, but mainly in the mornings to match the period of the day in which baseline examinations (including HRV recordings) took place.

Sociodemographic factors and previous medical history were gathered by interviews with questions about age, sex, self-declared ethnicity, educational level, self-reported smoking status, and physical leisure activity. Women were questioned regarding their menopausal status and whether they were under hormone-replacement therapy. Height and weight were measured using standard equipment and techniques, and the body mass index (BMI) was calculated as weight divided by height in meters squared. Blood pressure (BP) was measured three times using a validated Omron HEM 705CPINT oscillometric device at one-minute intervals. The mean of the two last BP measurements was considered the clinical BP. Hypertension was defined as using medication to treat hypertension or systolic blood pressure ≥140 mmHg and/or diastolic blood pressure ≥90 mm Hg. Dyslipidemia was defined by LDL >130 mg/dL or if the participant used any lipid-lowering medication.

Continuous variables were reported as means±SD or median and interquartile range (IQR), and categorical variables were presented as absolute numbers and proportions. The chi-squared, Student *t*-, and Wilcoxon tests were used whenever applicable, according to data distribution. We fitted three binary logistic regression models to determine whether dichotomized HRV indices (below and above the median value), as the independent variable, were associated with the dependent variable CAC score (CAC score>0 *vs* CAC score=0). Model I was adjusted for age, sex, and ethnicity; Model II was fully adjusted, that is, for variables in Model I plus, educational level, BMI, hypertension, diabetes, dyslipidemia diagnoses, statin use, physical activity level, smoking status, and time between HRV assessment and CT scan. We additionally performed subgroup analyses by age below and above 49 years, the median age of the sample.

To examine possible effect modification by sex in the association between CAC score and HRV indices, we performed additional analysis by including a multiplicative term between HRV indices and sex using the fully adjusted Model II. Then, age-stratified (<49 and ≥49 years) logistic regressions were performed for men *vs* women for associations between CAC and HRV measures. To account for menopausal status, we performed an additional model in age-stratified women controlling for menopausal and hormonal therapy status. Statistical analyses were performed using R software (R Core Team, Austria), version 3.6.3. The significance level was set at 0.05.

## Results

From the 4549 ELSA-Brasil participants who underwent computed tomography exams in the Sao Paulo site, 3940 had valid R-R interval time series that passed the quality control for HRV analyses. We excluded 171 individuals for previous cardiovascular disease (acute myocardial infarction, heart failure, cardiac surgery, and stroke), 248 for use of beta-blocker medication, 381 for use of other medications that could interfere HRV measures (ACE inhibitor, calcium channel blockers, and antiarrhythmic drugs), 1 for presenting arrhythmia during ECG recording (atrial fibrillation or atrial flutter), and 1 for use of an external cardiac pacemaker.

After exclusions, our final analytical sample consisted of 3138 participants, and Table 1 displays the sample characteristics at baseline. Prevalent CAC was observed in 765 (24.4%) participants, of which 535 (17.0%) had a CAC score between 0.1 and 99.9, 168 (5.4%) had a CAC score between 100 and 399.9, and 62 (2.0%) had a CAC score ≥400 Agatston units. In bivariate analyses, participants with prevalent CAC were older, predominantly men, with higher BMI, SBP, DBP, and had higher prevalence of hypertension, diabetes, dyslipidemia, and sedentarism.

**Table 1:** Study sample characteristics according to the prevalence of CAC.

Variable	Total (n=3138)	CAC score=0 (n=2373)	CAC score>0 (n=765)	P-value
Age (years)^a^	49.66 (8.28)	47.73 (7.32)	55.63 (8.23)	<0.001
Female sex (n, %)	1727 (55.0)	1452 (61.2)	275 (35.9)	
Ethnicity (n, %)				<0.001
White	1848 (58.9)	1367 (57.6)	481 (62.9)	
Brown	684 (21.8)	537 (22.6)	147 (19.2)	<0.001
Black	406 (12.9)	339 (14.3)	67 (8.8)	
Other/Missing	200 (6.4)	130 (5.5)	70 (9.2)	
Educational level (%)				<0.001
<High school	419 (13.4)	287 (12.1)	132 (17.3)	
High school	1303 (41.5)	1063 (44.8)	240 (31.4)	
College or above	1416 (45.1)	1023 (43.1)	393 (51.4)	
BMI (kg/m^2^)^b^	26.4 (23.8, 29.5)	26.2 (23.6, 29.4)	26.9 (24.3, 29.7)	0.002
SBP (mmHg)^b^	115.5 (106.5, 126.0)	114 (105.5, 124.0)	121 (111.5, 131.0)	<0.001
DBP (mmHg)^b^	73.5 (67.0, 80.5)	72.5 (66.5, 79.5)	76.0 (69.0, 83.0)	<0.001
Hypertension (n, %)	528 (16.8)	329 (13.9)	199 (26.0)	<0.001
Diabetes (n, %)	387 (12.3)	241 (10.2)	146 (19.1)	<0.001
Dyslipidemia (n, %)	1717 (54.7)	1205 (50.8)	512 (66.9)	<0.001
Current smokers (n, %)	537 (17.1)	363 (15.3)	174 (22.7)	<0.001
Statin use (n, %)	232 (7.4)	127 (5.4)	105 (13.7)	<0.001
Physical activity (n, %)				<0.001
Sedentary and insufficiently active	2315 (73.1)	1736 (73.1)	559 (73.0)	
Active	712 (22.7)	523 (22.0)	189 (24.7)	
Post-menopausal women, no hormonal replacement (n, %)	483 (15.4)	353 (14.9)	130 (17.0)	<0.001
Post-menopausal women, with hormonal replacement (n, %)	289 (9.2)	203 (8.6)	86 (11.2)	<0.001

CAC: coronary artery calcium; BMI: body mass index; SBP: systolic blood pressure; DBP: diastolic blood pressure. ^a^Mean±SD; ^b^Median and interquartile range. The chi-squared, Student *t*-, and Wilcoxon tests were used when applicable.

**Figure 1:**
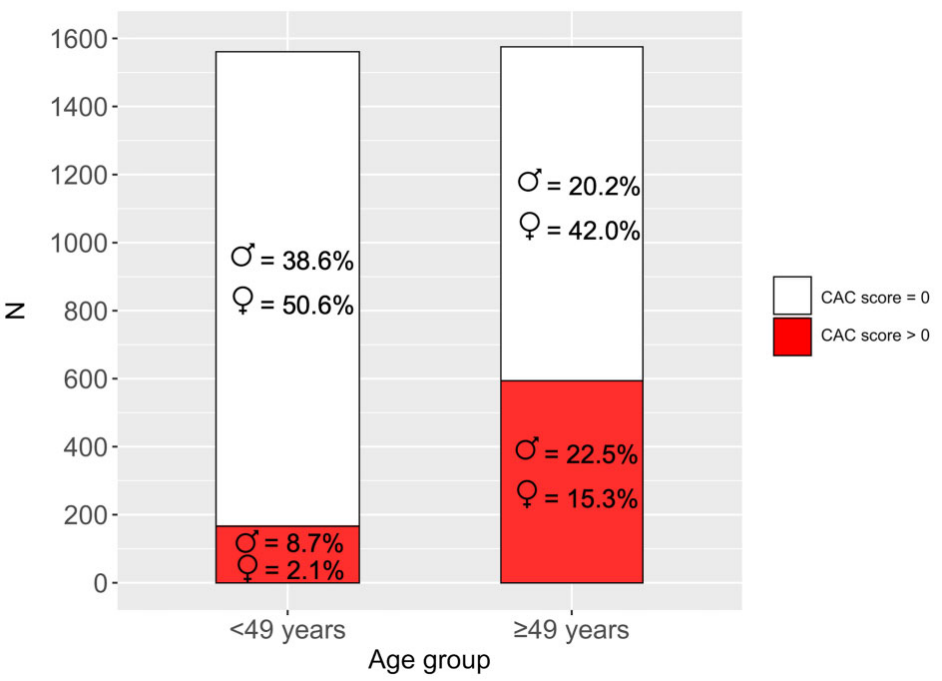
Frequency of coronary artery calcium (CAC) score in younger and older participants. The frequency of men in the indicated group is represented by the ♂ symbol, and the ♀ symbol represents women. Frequencies sum 100% in each bar.

Figure 1 displays the proportion of participants with prevalent CAC according to age groups (below and above 49 years, the median age for the sample). Prevalent CAC was observed in 596 (37.8%) among 1576 older individuals (≥49 years) and in 169 (10.8%) younger individuals (<49 years). Almost 40% of older participants with prevalent CAC presented low values for all four HRV indices *vs* 21.3% in the younger group.

**Figure 2:**
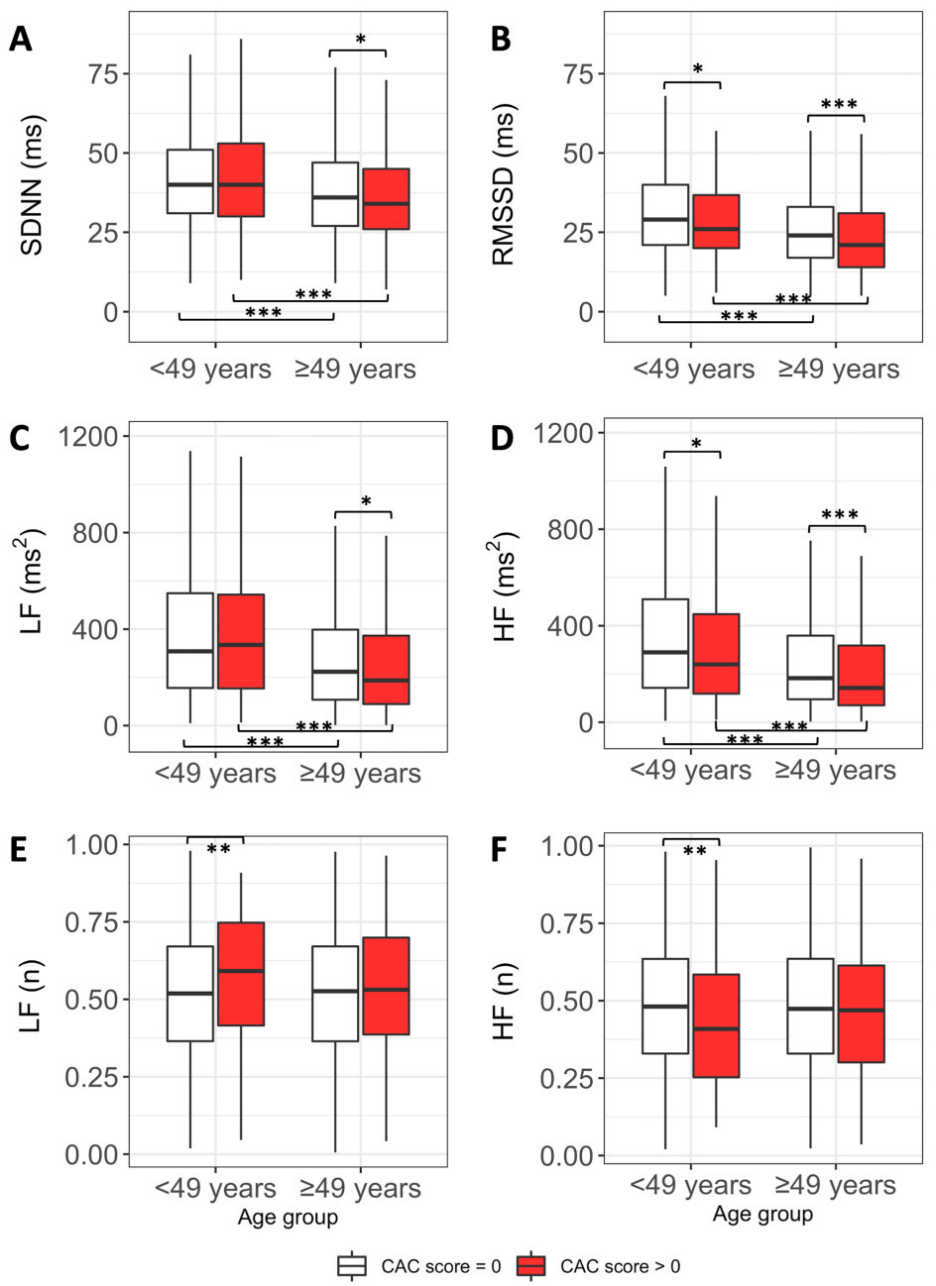
Distribution of heart rate variability (HRV) indices in age-stratified subgroups. CAC: coronary artery calcium; SDNN: standard deviation of NN interval; RMSSD: root mean square of successive differences between adjacent NN intervals; LF: low-frequency; HF: high-frequency. Data are reported as medians, interquartile ranges, and lower and upper limits. *P<0.05, **P<0.01, and ***P<0.001 for pairwise contrasts using Wilcoxon test and Holm-Bonferroni correction for multiple comparisons.

Tables 2 and 3 display the HRV profile of the sample consistently showing that individuals with prevalent CAC had significantly lower HRV values. Analyzing HRV distribution in age subgroups (Figure 2), we observed that this difference was mostly concentrated within older participants. RMSSD and HF were lower in prevalent CAC in both of age groups. Additionally, HRV values within both CAC score >0 and CAC score=0 groups were lower in older participants than in younger counterparts.

**Table 2:** Distribution of heart rate variability indices in the overall sample and in sex-stratified subgroups.

Overall sample	Total (n=3138)	CAC score=0 (n=2373)	CAC score>0 (n=765)	P-value
SDNN (ms)	38.0 (29.0, 50.0)	39.0 (30.0, 50.0)	35.0 (27.0, 48.0)	<0.01
RMSSD (ms)	26.0 (18.0, 37.0)	27.0 (19.0, 38.0)	22.0 (15.0, 33.0)	<0.01
LF (ms^2^)	279.0 (135.0, 554.0)	294.0 (144.0, 574.0)	228.0 (105.0, 479.0)	<0.01
HF (ms^2^)	242.0 (112.0, 516.8)	266.0 (128.0, 549.0)	170.0 (84.0, 416.0)	<0.01
LF (n)	0.53 (0.37, 0.68)	0.52 (0.37, 0.67)	0.55 (0.39, 0.71)	0.002
HF (n)	0.47 (0.32, 0.63)	0.48 (0.33, 0.63)	0.45 (0.29, 0.61)	0.002

Men	Total (n=1411)	CAC score=0 (n=921)	CAC score>0 (n=490)	P-value
SDNN (ms)	40.0 (30.0, 52.0)	41.0 (32.0, 53.0)	36.0 (27.0, 51.0)	<0.01
RMSSD (ms)	25.0 (18.0, 36.0)	27.0 (19.0, 38.0)	22.0 (15.0, 33.0)	<0.01
LF (ms^2^)	361.0 (171.5, 675.0)	413.0 (199.0, 736.0)	269.0 (123.5, 566.8)	<0.01
HF (ms^2^)	216.0 (103.5, 468.0)	244.0 (118.0, 509.0)	163.0 (77.8, 387.0)	<0.01
LF (n)	0.61 (0.47, 0.74)	0.62 (0.47, 0.74)	0.61 (0.45, 0.75)	0.677
HF (n)	0.39 (0.26, 0.53)	0.38 (0.26, 0.53)	0.39 (0.25, 0.55)	0.677

Women	Total (n=1727)	CAC score=0 (n=1452)	CAC score>0 (n=275)	P-value
SDNN (ms)	36.0 (28.0, 47.5)	36.0 (28.0, 48.0)	34.0 (26.0, 44.0)	0.02
RMSSD (ms)	26.0 (18.0, 37.0)	27.0 (19.0, 38.0)	23.0 (15.0, 33.0)	<0.01
LF (ms^2^)	233.0 (118.0, 442.5)	248.0 (124.0, 458.8)	169.0 (75.5, 346.0)	<0.01
HF (ms^2^)	264.0 (125.0, 558.5)	277.0 (133.0, 578.0)	193.0 (87.0, 440.5)	<0.01
LF (n)	0.46 (0.32, 0.62)	0.47 (0.32, 0.62)	0.45 (0.32, 0.62)	0.717
HF (n)	0.54 (0.38, 0.68)	0.53 (0.38, 0.68)	0.55 (0.38, 0.68)	0.717

Data are reported as median and interquartile ranges. The Wilcoxon test was used. CAC: coronary artery calcium; SDNN: standard deviation of NN interval; RMSSD: root mean square of successive differences between adjacent NN intervals; LF: low-frequency; HF: high-frequency; ms: milliseconds; ms^2^: milliseconds square; n: normalized units.

**Table 3:** Distribution of heart rate variability indices in sex- and age-stratified subgroups.

Men <49 years	Total (n=739)	CAC score=0 (n=603)	CAC score>0 (n=136)	P-value
SDNN (ms)	43.0 (33.0, 55.0)	43.0 (34.0, 54.0)	42.0 (31.0, 56.0)	0.681
RMSSD (ms)	29.0 (21.0, 41.0)	29.0 (21.0, 41.0)	26.5 (20.0, 37.3)	0.271
LF (ms^2^)	467.0 (228.5, 826.5)	483.0 (229.5, 829.0)	449.0 (221.0, 765.3)	0.471
HF (ms^2^)	295.0 (139.0, 567.5)	299.0 (137.5, 574.5)	282.0 (143.8, 520.3)	0.499
LF (n)	0.62 (0.47, 0.74)	0.62 (0.47, 0.74)	0.64 (0.47, 0.76)	0.553
HF (n)	0.38 (0.26, 0.53)	0.38 (0.26, 0.53)	0.36 (0.24, 0.53)	0.553

Women <49 years	Total (n=823)	CAC score=0 (n=790)	CAC score>0 (n=33)	P-value
SDNN (ms)	38.0 (30.0, 49.0)	38.0 (30.0, 49.0)	36.0 (30.0, 42.0)	0.131
RMSSD (ms)	30.0 (22.0, 40.0)	30.0 (22.0, 40.0)	25.0 (20.0, 32.0)	0.023
LF (ms^2^)	274.0 (142.0, 497.5)	278.5 (143.0, 500.8)	156.0 (101.0, 323.0)	0.01
HF (ms^2^)	337.0 (170.0, 646.0)	351.5 (171.3, 649.8)	223.0 (112.0, 378.0)	0.022
LF (n)	0.45 (0.31, 0.60)	0.45 (0.31, 0.60)	0.45 (0.35, 0.57)	0.893
HF (n)	0.55 (0.40, 0.69)	0.55 (0.40, 0.69)	0.55 (0.43, 0.65)	0.893

Men ≥49 years	Total (n=607)	CAC score=0 (n=274)	CAC score>0 (n=333)	P-value
SDNN (ms)	36.0 (27.0, 49.0)	39.0 (29.0, 50.0)	34.0 (26.0, 47.0)	0.04
RMSSD (ms)	22.0 (16.0, 31.0)	24.0 (17.0, 32.0)	21.0 (14.0, 30.0)	0.07
LF (ms^2^)	260.0 (130.0, 528.5)	309.0 (160.0, 559.0)	233.0 (109.0, 492.0)	0.02
HF (ms^2^)	158.0 (79.5, 340.5)	168.0 (96.0, 358.8)	142.0 (67.0, 315.0)	0.01
LF (n)	0.61 (0.45, 0.74)	0.62 (0.46, 0.74)	0.59 (0.45, 0.74)	0.423
HF (n)	0.39 (0.26, 0.55)	0.38 (0.26, 0.54)	0.41 (0.26, 0.55)	0.423

Women ≥49 years	Total (n=821)	CAC score=0 (n=586)	CAC score>0 (n=235)	P-value
SDNN (ms)	35.0 (26.0, 45.0)	35.0 (26.0, 45.0)	34.0 (25.0, 45.0)	0.487
RMSSD (ms)	23.0 (16.0, 33.0)	23.0 (17.0, 34.0)	23.0 (15.0, 33.0)	0.235
LF (ms^2^)	187.0 (87.0, 378.0)	199.0 (92.3, 382.8)	169.0 (72.0, 361.0)	0.037
HF (ms^2^)	198.0 (93.0, 449.0)	202.0 (98.3, 449.5)	181.0 (85.5, 446.0)	0.325
LF (n)	0.48 (0.32, 0.64)	0.49 (0.33, 0.64)	0.45 (0.31, 0.63)	0.214
HF (n)	0.52 (0.36, 0.68)	0.51 (0.36, 0.67)	0.55 (0.37, 0.69)	0.214

Data are reported as median and interquartile ranges. The Wilcoxon test was used. CAC: coronary artery calcium; SDNN: standard deviation of NN interval; RMSSD: root mean square of successive differences between adjacent NN intervals; LF: low-frequency; HF: high-frequency; ms: milliseconds; ms^2^: milliseconds square; n: normalized units.

**Figure 3:**
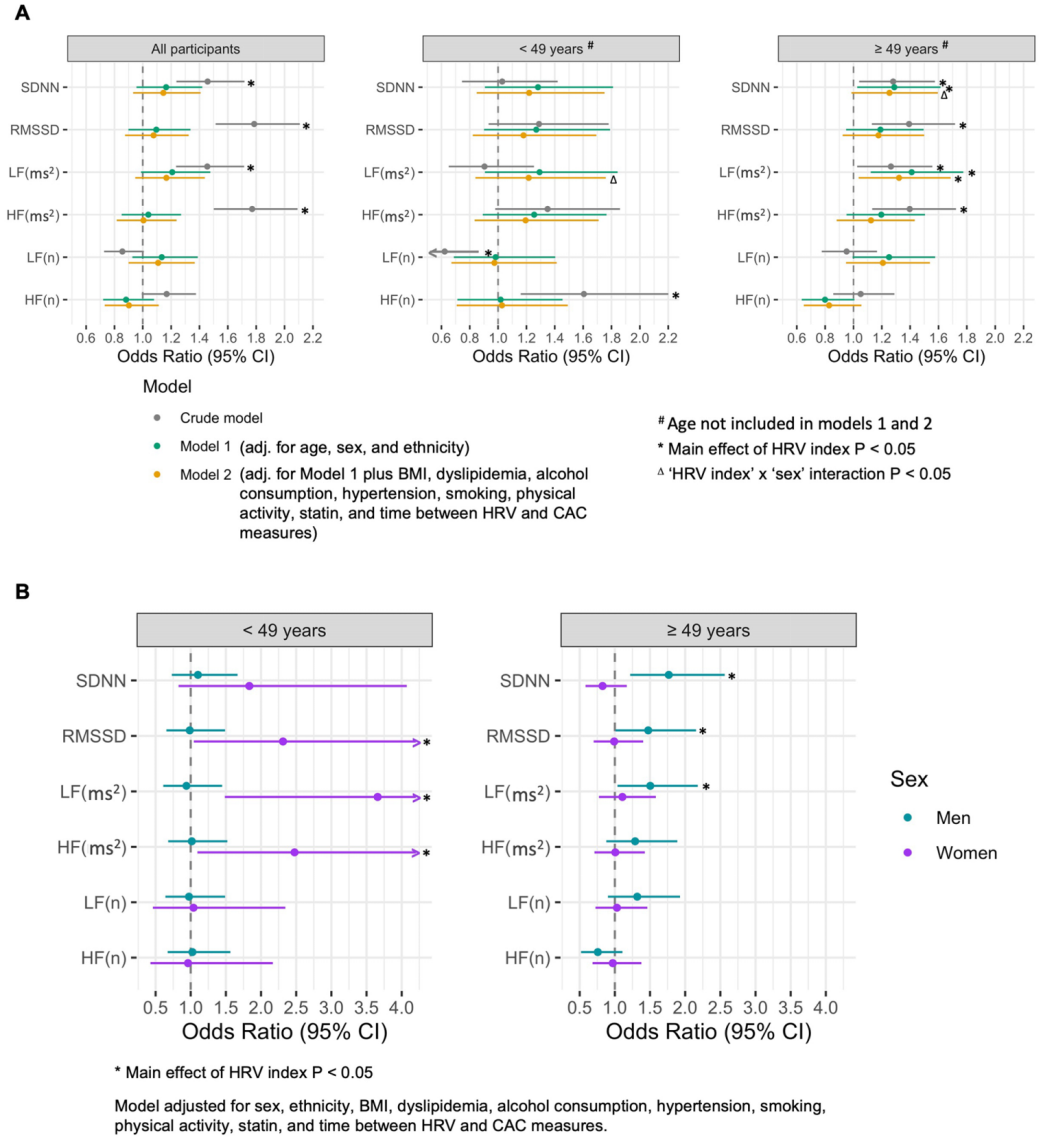
Logistic regression for associations between coronary artery calcium (CAC) and heart rate variability (HRV) measures in the overall sample and in age-stratified subsamples (<49 and ≥49 years old). **A**, Model I (green): adjusted for age, sex, and ethnicity. Model II (dark yellow): adjusted for Model I covariates plus body mass index (BMI), dyslipidemia, alcohol consumption, hypertension, smoking, physical activity, statin, and time between HRV and CAC measures. Error bars indicate 95%CI. *P<0.05 for HRV index main effect. ^Δ^P<0.05 for the interaction between HRV index and sex. **B**, Men (teal) *vs* women (purple) age-stratified (<49 and ≥49 years old) logistic regressions for associations between CAC and HRV measures using Model II. *P<0.05 for HRV index main effect. Error bars indicate 95%CI.

In the older group, a CAC score >0 was 32% (OR=1.32, 95%CI: 1.04-1.69) more likely in participants with lower LF values, regardless of sociodemographic and clinical factors, and 29% more likely in those with lower SDNN (OR=1.29, 95%CI: 1.03-1.62; Figure 3A), controlling for sex and ethnicity. Interaction analysis between HRV indices and sex in younger participants revealed significant effect modification for LF. Younger women with lower LF values were three-times more likely to present prevalent CAC (3.03, 95%CI: 1.31-7.00), while no significant association was observed in younger men (OR=0.94, 95%CI: 0.61-1.45; Figure 3B). Contrastingly, in the older group, interaction analysis showed significant results for SDNN (Figure 3A), with a significantly higher risk in men (OR=1.77, 95%CI: 1.22-2.56; Figure 3B). Finally, to account for menopausal status, we performed an additional model in women controlling for menopausal and hormonal therapy status, with no change in results.

## Discussion

To our knowledge, this is the first study to examine the association between cardio-autonomic markers and CAC scores in a large cohort of adults. Of note, the ELSA-Brasil cohort is a highly admixed and multiethnic sample, which was not selected from a particular group according to a specific disease or condition.

Our main finding was a significant association between LF and CAC in participants aged 49 years or older, regardless of sex, age, and other clinical factors. The LF power is a measure of vagal and sympathetic modulation. It also reflects baroreflex regulation of blood pressure and vasomotor tone during resting conditions (29). As the vasomotor system modulation oscillations (frequencies around 0.1 Hz) are within the LF spectral band (0.04-0.15 Hz), the expression of these perturbations on heart rate through a resonance phenomenon has been suggested to be one of the sources of the LF spectrum (30). Therefore, our results seem to align with the physiologic aspects related to LF index, suggesting an association between reduced parasympathetic and sympathetic activity, potentially with some level of baroreflex activity impairment, and CAC score >0.

Lower SDNN values in older participants, especially in men, as sex was an important effect modifier in this age group, was also associated with prevalent CAC. SDNN reflects global variability between adjacent heartbeats, and lower values have been associated with high levels of inflammation either in participants with no apparent heart disease (31) or in cardiovascular conditions, such as angina pectoris (32) and decompensated heart failure (33).

Previous cross-sectional and longitudinal studies examined this association in selected groups with type I diabetes, comparing the results to non-diabetic participants (20,21,34). Colhoun et al. (34) verified that significant unadjusted associations between HRV total spectral power and CAC in all participants combined or groups (type I diabetics and controls) did not maintain the significance level after adjustments, similar to our results in the entire sample. Rodrigues et al. (20) reported that reduced HRV predicted the progression of CAC in adults with and without type I diabetes, independently of known cardiovascular disease risk factors or inflammatory markers. Contrastingly, Hjortkjær et al. (21) found no evidence of cardiovascular autonomic neuropathy being a risk factor for progression of CAC in patients with type 1 diabetes. More similar to our sample but using a different approach, Jae et al. (35) observed that slower postexercise heart rate recovery (HRR), a cardiovagal function marker, was associated with advanced CAC (>75th percentile according to age) in healthy participants. Based on previous findings and our results, we hypothesize that there may be a participation at some extension of autonomic neuropathy in the pathophysiological processes of atherosclerosis with inflammatory involvement in adults.

In a recent article, we detected lower HRV values in participants with high carotid intima-media thickness (cIMT), but independent relationships were not found in the overall sample (36). It is important to note that these different findings may be related to CAC and cIMT distinct performances in relation to subclinical atherosclerosis progression: cIMT reflects the first stages while CAC reflects later stages of the atherosclerotic process (37). Our results also showed that sex influenced the relationship between HRV and CAC score status within age groups. Specifically, decreased LF index showed increased OR in younger women. This is particularly relevant because although low HRV and prevalent CAC were not associated in overall younger individuals, women in the younger group were more prone to CAC score >0 when this spectral index was reduced. This relationship was significant even though only 4% of younger women had prevalent CAC (which explains the large confidence intervals). Estrogen has been reported to enhance the activity of choline uptake and acetylcholine synthesis, the primary neurotransmitter released by the parasympathetic nervous system (38). Our hypothesis is that the ANS plays such an important role in arterial calcification that impaired activity overrides female hormonal cardioprotection.

It is well documented that aging progressively increases sympathetic activity and decreases parasympathetic modulation (28,39), causing detrimental effects on endothelial and vascular function through vascular wall innervation. Sympathetic hyperactivation leads to vasoconstriction, loss of vascular elasticity, accumulation of modified lipoproteins in the vascular wall. It also increases peripheral vascular resistance, induces endothelial dysfunction, stimulates oxidative stress, and vascular remodeling, and favors micro- and macro-calcification in both the vascular intima and media (18,19). This autonomic condition also favors pro-inflammatory and prothrombotic effects (19) driven by cytokines, chemokines, and other biologically active mediators (16,17). The concept of an inflammation-dependent calcification paradigm suggests macrophage infiltration and inflammation preceding calcification. In this sense, inhibited anti-inflammatory pathways and anti-apoptotic effects caused by reduced vagal control may facilitate the formation of microcalcifications in vascular walls that can be mediated by cell death and release of apoptotic bodies, which content is similar to vesicles found in the physiological mineralization of the bone (18).

The common co-existence of ANS abnormality and endothelial dysfunction suggests interactions between them, which may be involved in the pathogenesis of different cardiovascular diseases (19). The intimate relationship of sympathetic and parasympathetic nervous systems and vascular walls through the innervation chain supports the putative pathway linking the presence of cardio-autonomic dysfunction to a higher atherosclerotic burden (18). Nonetheless, the pathways that mediate ANS effects on vascular structure and function, and vice-versa, are complex and need to be fully determined and characterized. The rationale for investigating whether HRV is associated with preclinical coronary atherosclerosis resides in the fact that it is a potential marker for higher cardiovascular risk and may be proven helpful to identify individuals who warrant further evaluation and specific exams, such as a CT scan. Furthermore, as atherosclerosis is a dynamic process continually changing and morphing, future research can explore whether proportional changes in HRV accompany modifications in CAC.

The main strength of our study is that we analyzed CAC data from a large sample outside the United States and Europe. This cohort consists of civil servants who volunteered to participate and were not selected from inpatient/outpatient clinics, or specialized services, ruling out selection bias by disease.

As aforementioned, ANS control and vascular function interact reciprocally (18,19); therefore, the cross-sectional design of this study does not allow causality assumptions. To minimize the problem of reverse causality, we excluded participants with previously diagnosed cardiovascular disease from the analysis. Additionally, although CAC score correlates with the extent of coronary atherosclerosis and is a good predictor of cardiovascular risk, it cannot detect non-calcified atherosclerotic plaques. Nonetheless, this situation is expected to be rare and not significantly change the results in samples without previous or suspected coronary heart disease (low pre-test probabilities of coronary disease).

In summary, we verified decreased HRV in the presence of CAC score >0 and an association between these markers in a sample without major cardiovascular disease. Specifically, individuals with lower LF values were more likely to have CAC score >0 in participants aged 49 years or older, independent of sociodemographic and clinical factors. Lower SDNN values were also associated with prevalent CAC in older participants independent of sex and ethnicity. Specifically regarding sex, younger women with decreased LF and older men with low SDNN were more likely to have prevalent CAC. These findings suggest that the association between CAC and some HRV indices might be sex- and age-specific. Furthermore, these results may reflect complex interactions between the autonomic and vascular systems, which can be involved in the pathogenesis of atherosclerosis.
